# Liver Stiffness Evaluation in Chronic Hepatitis C Patients with Cirrhosis before and after Direct-Acting Antivirals

**DOI:** 10.3390/microorganisms12071418

**Published:** 2024-07-12

**Authors:** Cristina Stasi, Stefano Brillanti

**Affiliations:** Department of Medicine, Surgery and Neuroscience, University of Siena, 53100 Siena, Italy

**Keywords:** hepatitis C virus, chronic hepatitis C, liver cirrhosis, liver stiffness, non-invasive evaluation, transient elastography

## Abstract

After the introduction of direct-acting antivirals, parallel significant clinical progress has been achieved in the assessment of liver fibrosis progression/regression before treatment and during the follow-up of the cirrhotic patients with chronic hepatitis C virus (HCV) infection. The evolution of chronic hepatitis C into liver cirrhosis is correlated with an extensive accumulation of the extracellular matrix, leading to the formation of large amounts of fibrotic tissues that, initially, are concentrated in periportal areas and, in the later stages, surround the nodules of regenerating hepatocytes. The progressive increase in the fibrotic matrix contributes to vascular disturbances (favoring the development of portal hypertension) and to microenvironmental changes. The four clinical stages of liver cirrhosis are predictors for different clinical scenarios. The wide-ranging functions of the liver require different methods for their assessment. The non-invasive evaluation using transient elastography is useful in determining the longitudinal modifications of fibrosis during and after treatment with direct-acting antivirals. The liver stiffness evaluation, known to have a wide range of values in cirrhotic patients, can offer different prognostic implications after sustained virological response. This review discusses the different time points of liver stiffness evaluation that appear to show a more well-defined propensity to identify adequate monitoring schedules for these patients.

## 1. Introduction

The prevalence of chronic liver diseases is increasing worldwide. According to data from the Global Burden of Disease, liver cirrhosis is among the top 10 causes of death in the world, accounting for about 160 million people suffering from cirrhosis worldwide. Globally, cirrhosis mortality cases have increased by 47.15% from 1990 to 2017. As of 2017, the most frequent causes of mortality burden of liver cirrhosis are chronic hepatitis B virus infection (HBV, 29.03%) and hepatitis C virus infection (HCV, 25.9%), followed by alcoholic cirrhosis, other causes of cirrhosis, and non-alcoholic steatohepatitis (NASH) [1]. In 2021, the annual number of deaths from cirrhosis and other chronic liver diseases was estimated to be 1,425,142 [2].

Liver cirrhosis is an evolving process of all chronic inflammatory and/or degenerative pathologies (wounding response) that leads to a massive proliferation of connective tissue in the liver parenchyma. This clinical syndrome results from exogenous/toxic causes, viral infections (HCV, HBV), immunopathological/autoimmune causes such as primary biliary cholangitis, primary sclerosing cholangitis, and autoimmune hepatitis, vascular processes or inborn errors of metabolism, and hereditary diseases, such as hemochromatosis and Wilson’s disease [3].

Hepatic cirrhosis is characterized by the presence at portal spaces of fibrous (active and passive) septa of both the porto-portal and porto-central areas, with a parallel presence of regenerative nodules that subvert the normal hepatic architecture. This remodeling can result in organ collapse characterized by the massive necrosis/apoptosis of parenchymal cells and vascular disruption such as the capillarization of sinusoids and the formation of intrahepatic shunts, closely associated with angiogenesis defined by the formation of new vessels deriving from those already existing. These alterations are already present in the early stage of the disease but remain unnoticed for a long time. Based on these premises, this review discusses the different time points of liver stiffness evaluation that appear to show a more well-defined propensity to identify adequate monitoring schedules for these patients.

## 2. Clinical Assessment of Liver Cirrhosis

Liver cirrhosis can be classified into two phases, compensated and decompensated, which in turn are divided into four stages (1° and 2° in compensated and 3° and 4° in decompensated). During the compensated phase, no symptoms occur and the portal pressure is still below the limit for the onset of cirrhosis-related complications. The decompensated phase is specifically identified by the development of two stages (3° and 4°) based on developed specific complications. In the phase of hepatic decompensation, portal pressure increases, and patients are at risk of developing several outcomes (ascites, bleeding from esophageal varices, sepsis, especially spontaneous bacterial peritonitis, encephalopathy, non-obstructive jaundice, and hepatocellular carcinoma). Each of these is characterized by a different mortality rate. Briefly, as reported by D’Amico et al. [4], in Stage 1 (patients without esophageal varices and ascites) the mortality rate is about 1% per year; in Stage 2 (patients with esophageal varices without ascites and bleeding) the mortality rate is about 3.4% per year; in Stage 3 (patient with ascites with or without esophageal varices that have never bled) the mortality rate is about 20% per year; in Stage 4 (gastrointestinal bleeding with or without ascites) the mortality rate is about 57% for one year. When the decompensated disease is associated with major complications, the Child–Pugh, MELD, and ALBI scores are useful in determining the severity of liver cirrhosis and its clinical implications [5,6,7,8,9]. Alternative fibrosis staging systems, such as evaluating hepatic stiffness with a wide range of values in cirrhotic patients, can detect a different prognostic evaluation after direct-acting antiviral agents [10].

Esophagogastroduodenoscopy is a widely recognized method to detect gastroesophageal varices and evaluate the risk of bleeding.

Transient elastography (TE), or Fibroscan^®^, is a method used to measure liver stiffness. In Italy, the most used cut-offs for liver fibrosis corresponding to the METAVIR score were F3 > 10 kPa, and F4 > 13 kPa [11]. The meta-analysis by Tsochatzis et al. [12] suggests that the average optimal liver stiffness value (LSV) for cirrhosis is the cut-off of 15 ± 4.1 kPa—median 14.5—values between 9.0 and 26.5 kPa. In liver cirrhosis, the ultrasound reveals a lack of homogeneity in the liver tissue, an irregular liver surface, and an enlargement of the caudate and left lobe. The ultrasound can identify signs of portal hypertension, such as a spleen bipolar diameter of >12 cm or the largest splenic cross-sectional area passing through the hilum of >45 cm^2^, and a reduced portal vein blood flow velocity (time-averaged mean velocity of <14–16 cm/s^2^); it can also detect the presence of ascites [13,14].

The recent Baveno VI criteria [15] suggests endoscopic screening in patients with platelets < 150,000 and stiffness > 20 kPa to exclude the presence of varices at high risk of bleeding and, therefore, to select patients who can safely avoid the screening with esophagogastroduodenoscopy offered to all patients with newly diagnosed compensated cirrhosis.

Transient elastography and an ultrasound-based technique, the acoustic radiation force impulse (ARFI), play a fundamental role in studying liver fibrosis. They differ in the approaches used because TE employs an external mechanical push and the ARFI an internal acoustic one. These techniques measure the speed of shear waves in tissues that correlate to liver stiffness, expressed in kilopascals (kPa) [16]. Furthermore, portal hypertension leads to the enlargement of the elastic components of the spleen and subsequent splenomegaly. Liver stiffness is particularly useful for confirming existing liver cirrhosis or excluding it; therefore, clinicians should also monitor their patients for early liver cirrhosis screening. Recently, some studies have evaluated the utility of TE for evaluating short- and long-term longitudinal changes in liver fibrosis in patients with chronic HCV infection undergoing and who have undergone antiviral treatment with direct-acting antivirals (DAA). These research projects demonstrated that liver stiffness can also detect the longitudinal variation in liver fibrosis.

## 3. The Advancement and Revolution in the HCV Treatment

To date, the direct-acting antivirals’ effectiveness in the treatment of chronic HCV infection has oriented the scientific debate on issues relating to equity of access and the possible eradication of the virus.

About 10 years ago, therapies with pegylated interferon (Peg-IFN)-α (2a or 2b) in combination with ribavirin (RBV) were prevalent, which represented the “standard of care” for chronic infection from HCV. However, treatment with Peg-IFN-α and RBV had limited efficacy. Specifically, 48 weeks of therapy with Peg-INF and RBV achieved sustained virologic response (SVR) in 41.8% of patients with the chronic HCV infection genotype 1 [17]. Furthermore, these therapies were burdened by numerous relevant adverse events and were the cause of poor adherence and premature interruption of treatment. Patients who had had an absent or partial response to previous therapy with Peg-IFN and RBV or who had not been willing to take this type of therapy did not have alternative treatments available. This underlines that in those co-infected with HIV and those who had comorbidities with cardiac pathologies, chronic kidney disease, renal failure, kidney transplant, psychiatric pathologies, liver transplant and HCV-related liver disease, treatments with Peg-IFN and RBV may be contraindicated.

The introduction of DAAs has completely revolutionized the scenario of the treatment of HCV infection. At the beginning of the use of the first generation DAA, INF and RBV were used in combination with the new DAAs. In fact, treatment with the DAAs, telaprevir or boceprevir, administered with PegIFN and RBV, was suggested for a genotype 1 chronic HCV infection [18]. Many of the second-generation DAAs were then used INF-free. Interferon-free therapies are well tolerated and effective, but their initially high cost forced clinicians to follow recommendations to prioritize prescriptions.

In this new context, the primary goal of treating chronic HCV infection is to achieve SVR, defined as the absence of HCV RNA, at 12 and 24 weeks after the discontinuation of the treatment. These new regimes have a shorter duration of treatment and a good safety profile. DAAs are burdened by fewer side effects and provide high SVR rates.

The recommendations of the European Association for the Study of the Liver, published in 2015 [19], emphasized the goals of treating patients with HCV infection to prevent necroinflammation, fibrosis, cirrhosis, decompensated cirrhosis, hepatocellular carcinoma, severe extrahepatic disease manifestations and death. However, since not all patients with chronic HCV infection could be treated immediately, the EASL suggested giving priority to treatment based on the stage of fibrosis, risk of progression to more advanced disease, the presence of HCV-related extra-hepatic manifestations, and risk of HCV transmission (injecting drugs, sexual practices at high risk such as sexual intercourse between men, women of childbearing age who wish to become pregnant, patients on hemodialysis, and prisoners). Treatment was, therefore, a priority for patients with advanced fibrosis (Metavir with score F3–F4), including patients with decompensated cirrhosis, who had a contraindication to the use of IFN but could be treated with IFN-free regimens.

The new molecules can treat more than 90% of HCV-infected patients. In the AMBER RWE study, even in the population of patients with advanced liver disease, the effectiveness of the first-generation regimen (ombitasvir/paritaprevir/ritonavir ± dasabuvir ± ribavirin) reached 99% [20]. A more recent meta-analysis using data from 18 cohorts for a total of 12,531 subjects treated with glecaprevir/pibrentasvir demonstrated a SVR of 96%, and in those with compensated cirrhosis (six cohorts) of 97.8%. In these six cohorts, four patients who received glecaprevir/pibrentasvir had documented hepatic decompensation events [21]. The European Association for the Study of the Liver [22] underlined that given the new therapeutic options that allow the eradication of the infection, the emphasis of this branch of hepatology must be aimed at promoting the activation of screening, targeted diagnostic strategies, and ever-wider access to care.

Despite these highly effective treatments and the WHO recommendations that include the elimination of hepatitis by 2030, with a consequent reduction in infections by 90% and mortality by 65% by 2030 [23], 58 million people worldwide live with HCV, which causes approximately 400,000 deaths each year. According to WHO data, only 21% of the 58 million people with chronic hepatitis C had been diagnosed, while overall 13% had been treated in 2019 [24].

**Figure 1:**
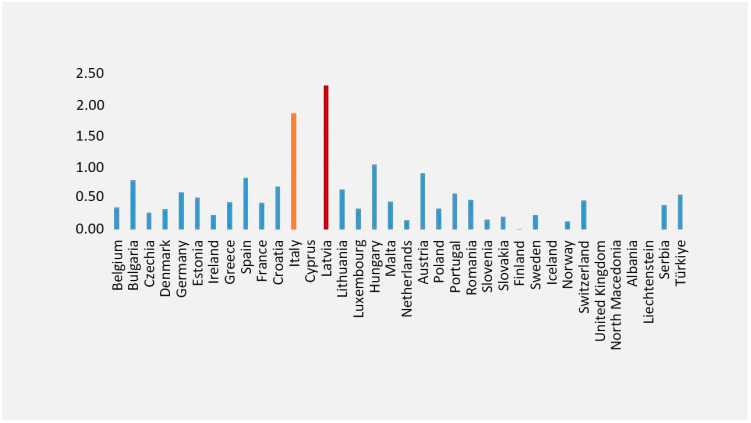
Mortality from viral hepatitis and sequelae of viral hepatitis. Legend: Standardized death rate due to hepatitis and sequelae of viral hepatitis (2021). This figure was realized based on our elaboration of Eurostat data [25]. For some countries, data were not available [25] The highest death rates from viral hepatitis and sequelae of viral hepatitis were in Latvia (in red) and Italy (in yellow).

According to Eurostat data, Italy ranks second (Figure 1) for the highest mortality rate from viral hepatitis among EU Member States, with a death rate of 1.85 from viral hepatitis and the sequelae of viral hepatitis [25].

Based on GBD 2017 Cirrhosis Collaborators, Latvia is one of the eight countries (Lithuania, Ukraine, Belarus, Russia, Kazakhstan, Estonia, Latvia, and Armenia) with a substantial increase in the cirrhosis age-standardized death rate, and where the majority of deaths in 2017 were due to alcohol-related liver disease [26].

In this regard, an Italian study [27] conducted through the analysis of data from the National Register of Causes of Death, highlighted that in 1.6% of deaths occurring in people aged ≥ 20 years, HCV infection is present (corresponding to 27,730 deaths).

The mortality rate associated with HCV infection increases exponentially with age in both sexes and is higher in Southern Italy, with the highest peak among elderly people aged ≥ 60 years.

## 4. Longitudinal Evaluation of Liver Stiffness in HCV-Related Cirrhosis before and after DAA Treatment

Several studies have evaluated the utility of elastography and non-invasive markers for the assessment of longitudinal changes in liver fibrosis and as a predictor of events in HCV-infected patients before and after antiviral treatment [28,29,30,31].

Sirinawasatien et al. [32] evaluated liver fibrosis with surrogate markers of liver fibrosis (indirect markers of fibrosis and LSV) 24 weeks after the end of DAA treatment, with the primary outcome of improvement > 30% in LSV. Out of 110 chronic HCV patients (median LSV at baseline = 15.05 kPa), 28.2% showed significant fibrosis (LSV between 7.0 and 9.4 kPa), 11.8% advanced fibrosis (LSV between 9.5 and 3.4 kPa) and 60% cirrhosis (LSV ≥ 13.5 kPa). The study demonstrated that a high LSV ≥ 9.5 kPa had a higher probability of meeting the primary outcome.

Berenguer et al. [33] in a large cohort of HIV/HCV patients (N = 1300) with advanced fibrosis or cirrhosis, achieving SVR from 2014 to 2017 in Spain, evaluated long-term clinical outcomes and prognostic factors for liver disease progression. The cohort was composed of 29.5% of patients with advanced fibrosis, 58.5% with compensated cirrhosis and 11.9% with decompensated cirrhosis. The study evaluated the risk of decompensation 1 year after DAA using the post-treatment LSV/platelet count criterion developed by Semmler and colleagues [34]. The incidence rate of decompensation person years was zero among patients (N = 206) with LSV < 12 kPa and platelet count > 150 × 10^9^ (low risk), while for patients in the grey zone or with LSV > 25 kPa (high risk), the incidence rates of decompensation were 0.36 (0.12–1.13) and 1.70 (0.55–5.28) per year, respectively. However, non-liver non-AIDS-related events were the main morbidity and mortality causes after DAA in advanced fibrosis/cirrhosis patients.

A recent interesting study by Nicoletti et al. [35] investigated changes in LSV in HCV cirrhotic patients undergoing DAA treatment, using two-dimensional shear wave elastography (2D-SWE) for predicting the occurrence of liver-related events before treatment and 24 and 48 weeks after end of treatment (EOT) during a median follow-up of 3.25 years. In this study, 229 patients enrolled were followed up every 6 months. The LSV of 18.1 (±6.6 SD) kPa at baseline decreased to 13.6 (±6.1) kPa at 6 months and to 12.5 (±6.1) kPa at 12 months with statistically significant differences among time points. During follow-up, 20.1% of patients reached an LSV < 10 kPa (cut-off for liver cirrhosis). The highest decrement of LSV was observed after 6 months from EOT compared to the reduction observed 12 months after EOT. The authors suggested the need for an appropriate reassessment of LSV cut-offs to evaluate liver fibrosis after DAA treatment. At univariate analysis, the development of ascites was associated with platelet count, INR, LSV and 1-year Delta LSV < 20%. At multivariate analysis, the occurrence of all portal hypertension-related events was associated with baseline LSV, portal velocity and 1-year Delta LSV < 20%.

A Single Center Cohort Study by Czarnecka et al. [36] on 51 HCV chronic kidney disease patients treated with DAA showed a significant LSV regression 4 years after SVR compared to baseline values (median LSV of 6.1 kPa vs. 4.9 kPa). Of these patients, only one presented advanced liver fibrosis at the baseline and progressed toward cirrhosis-based LSV measurements.

Rabell-Bernal et al. [37] measured the LSV using TE in Hispanic patients treated with DAAs after SVR. Out of 43 patients, 23.3% had F3 and 34.9% had F4 consistent with cirrhosis. At 6 and 9 months after reaching SVR, the fibrosis stage evaluated using LSV decreases in 20.9% of the patients with F4 and in 7.0% of those with F3. Only 30% of patients with advanced fibrosis (F3–F4) patients remained at their baseline stage.

An Italian retrospective study [38] analyzed data from 373 HCV-infected patients, of whom 94.4% had CHILD-A, treated with DAAs. The pre-treatment median LSV was 19.3 (14.7–27.0 kPa). A total of 80% of these patients presented portal hypertension and low hepatic synthesis. The LSV showed an overall improvement, reaching 11.6 (7.7–16.8 kPa) 6 months after SVR. When patients were divided by baseline platelets (lower than 150 × 10^9^/L) and albumin levels (lower than 3.5 g/dL), the LSV decreased by 1.6-fold. In CHILD B/C cirrhotic patients, the LSV decreased by 2-fold. These findings indirectly support not only the regression of liver fibrosis but also suggest the beneficial impact of DAA treatment on portal hypertension. Mezina et al. [39] investigated changes in LSV using TE in a large cohort (813 patients) of United States patients with chronic HCV, of whom 419 (52%) underwent DAA treatment. Treated patients were followed for 11.7 months and untreated patients for 12.7 months. Surprisingly, the results demonstrated no significant changes between LSV in treated versus untreated patients and in patients divided into cirrhotic (kPa ≥ 12, *n* = 119) and non-cirrhotic (kPa < 12, *n* = 651). The authors suggest that the apparent lack of improvement in LSV among treated patients may be due to other causes of liver injury (such as non-alcoholic fatty liver disease, alcohol use, and drug use) not fully included in the dataset. Furthermore, as some of the subjects treated had advanced fibrosis before treatment, there may still have been a progression of liver fibrosis. However, a higher LSV at the baseline was an independent predictor of a reduction in LSV in both groups. Contrary to what was observed by Mezina et al. [39], Alswat et al. [40] in a cohort of 172 HCV patients treated with DAA and reaching SVR found statistically significant differences at a follow-up of 141 (57.9) weeks compared to the baseline for LSV, APRI, FIB-4, and ALT/AST ratio in patients with F0-1 compared to those with >F2 fibrosis, supporting the post-treatment fibrosis regression, although in the study by Alswat et al. [38] the follow-up that was carried out was longer than that of Mezina et al. [39]. Davidov et al. [41] enrolled 133 SVR patients who had reached at least 6 months after EOT, of whom 61.7% had compensated for cirrhosis with a Child–Pugh score of A. In these patients, the liver fibrosis stage, assessed using a shear wave or FibroTest©, decreased by at least 1 stage in 56% of patients. In particular, the LSV evaluated using shear wave significantly improved during the first year after EOT and remained stable during the second year. Knop et al. [42] analyzed liver and spleen stiffness using elastography to evaluate the potential regression of cirrhosis and portal hypertension after DAA at different time points (24 weeks after EOT and 1, 2 and 3 years post-treatment). The LSV assessed using TE decreased between baseline values (median = 32.5 kPa) and EOT (21.3 kPa), and EOT and 3 years after (16 kPa), demonstrating a regression of LSV, but not of splenic stiffness. The authors therefore suggested that patients with advanced liver disease should undergo surveillance programs for complications of portal hypertension despite successful antiviral treatment and the regression of liver stiffness. Table 1 summarizes the main studies on liver fibrosis progression/regression in HCV patients after DAA treatment. Ferreira et al. [43] studied, in patients with chronic HCV infection, if the elimination of the virus after DDAs treatment alters the severity of liver disease (evaluated using LSV) and the metabolic/cellular profile. Out of 134 patients evaluated after DAA treatment, 82 patients presented a LSV = F1/F2 and 52 patients presented a LSV = F3/F4. The study demonstrated an overall baseline risk for higher fibrosis stages (F3/4) of 2.410. A significant decrease in stages of liver fibrosis was also observed after treatment (F3 = 9.7% at baseline vs. F3 = 8.2% after DAA treatment and F4 = 29.1% at baseline vs. F4 = 14.2% after DAA treatment). The authors found that the improvement of LSV was mainly correlated with higher baseline levels of platelet count and HDL and lower insulin resistance. Medrano et al. [44] evaluated change in LSV, hepatic venous pressure gradient (HVPG), Child–Pugh–Turcotte (CTP) score, and plasma biomarkers of bacterial translocation, inflammation, endothelial dysfunction, coagulopathy, and angiogenesis, 48 weeks after DAA treatment in 50 HIV-infected patients with HCV-related advanced cirrhosis. The study demonstrated an overall improvement after DAA treatment in the severity of advanced cirrhosis and plasma biomarkers. Notably, the study revealed that plasma biomarker levels were primarily associated with LSV reduction.

Despite the heterogeneity of the treatments used, both based on the availability of the regimens in the study period and in accordance with international guidelines on the treatment of cirrhotic patients, all patients achieved high percentages of SVR and a decrease in LSV. Regardless of the type of DAA regimen used, virus clearance is associated with decreased LSV due to both initially reducing necroinflammation and the subsequent regression of fibrosis.

Other prospective/retrospective studies have shown that high liver stiffness values may also have a negative predictive value on the occurrence/recurrence of hepatocellular carcinoma (HCC) [45,46,47,48,49]. Ravaioli et al. [50] retrospectively evaluated changes in the LSV as a predictor of the risk of HCC onset/relapse after DAA treatment in HCV cirrhotic patients, with a mean LSV at the baseline in the entire cohort (with and without development of HCC after treatment) of 18 kPa, followed-up for a median of 15 months. The results of this study highlighted a significantly lower decrease in LSV (−18%) in patients with the development of HCC after DAA treatment compared to LSV (28.9%) in those without the onset of HCC. Furthermore, a decrease in LSV of less than −30%, a CTP score of B, and a history of previous HCC were strongly associated with the risk of HCC onset/recurrence. Therefore, Ravaioli et al. [50] suggest the surveillance of cirrhotic patients with HCV by evaluating the LSV at EOT and calculating the LS variations to intercept a possible onset/recurrence of HCC after treatment with DAA [50]. Most of these studies consider an assessment of liver fibrosis before treatment to evaluate outcomes [32,46,47]. However, only a few studies have longitudinally evaluated the effect of DAAs in cirrhotic patients and HCC occurrence/recurrence in terms of sensitivity and specificity depicted by the Receiver Operator Characteristic (ROC) curve [50]. Interestingly, Pons et al. [46] evaluated outcomes after treatment. In fact, in this study, baseline LSV ≥20 kPa and low LSV improvement during the follow-up resulted in an increased risk of outcomes. In particular, during the follow-up, only albumin levels and LSV <10 kPa were predictors associated with the risk of presenting with HCC during follow-up. For an extensive review of LSV to predict the occurrence and recurrence of hepatocellular carcinoma before and after DAA treatment, see Stasi and Brillanti [51].

Based on clinical practice, this suggests interpreting the LSV after treatment with caution. The correlation between clinical and non-invasive methods could have a prognostic value. An accurate fibrosis assessment plays a critical role in guiding diagnosis, treatment, and prognostic assessment in liver cirrhosis. The various cirrhosis phases will probably have different outcomes after treatment, meaning that a better staging of cirrhosis phases could have prognostic significance. The identification of different phases of cirrhosis, characterized by its own cumulative risk of outcomes, including HCC occurrence/recurrence, could provide helpful information in the management of patients. The presence of ascites in the decompensated cirrhosis represents a limit for the liver stiffness evaluation using Fibroscan. Different staging systems for cirrhosis phases have been proposed, some of which have a defined prognostic value relative to survival. Some of these, such as Child–Pugh and MELD, had the prognostic value at 12 weeks after the EOT, not by baseline liver function. Since the use of DAA reduces the necessity of close follow-up monitoring and the number of resources required for disease progression, including end-stage liver disease, HCC, and the need for a liver transplant, it would be very interesting to clearly define the possible “point of no return” in cirrhotic patients at which the reversibility of fibrosis after DAA treatment does not change the prognosis, due to the pre-existing neoangiogenesis and liver regeneration that continue to support the evolution to hepatocellular carcinoma.

## 5. Conclusions

The prevalence of chronic liver disease is increasing, and its evolution into liver cirrhosis represents one of the leading causes of death worldwide. Consequently, accurate fibrosis assessment plays a critical role in the early identification of advanced fibrosis/cirrhosis. Furthermore, in cirrhotic patients, although the structural subversion linked to this condition continues to persist, the evaluation of post-treatment liver stiffness does not always correspond to the patient’s clinical picture due to the regression of liver stiffness. Therefore, this implies that, in some patients, stiffness values after DAA treatment should be considered with caution compared to those detected before treatment in dictating the time point of surveillance.

## Figures and Tables

**Table 1:** Main studies on cirrhotic HCV patients undergoing DAA treatment reporting liver stiffness values (before and after treatment).

Study Design	Cirrhotic Patients (F4)	DAA	Follow-Up	Country	Baseline LSV	Outcomes	References
Prospective cohort study	110, of whom 66 were cirrhotic patients	Cirrhosis was treated with SOF + LDV plus weight-based RBV (SOF + LDV + RBV) for 12 weeks	Over 24 weeks	Thailand	Median: 15.05 (8.76–23.68) kPa Cirrhosis (LSV ≥ 13.5 kPa), *n* (%)	Median: 9.60 (6.50–14.40) kPa at 24 weeks 52% of patients experienced a reduction of >30% in LSV over 24 weeks	Sirinawasatien et al., 2024 [32]
Multicenter retrospective study	384 advanced fibrosis; 761 compensated cirrhosis; 155 decompensated cirrhosis	Sofosbuvir/Ledipasvir or Sofosbuvir and Daclatasvir or Ombitasvir/Paritaprevir/Ritonavir and Dasabuvir or other direct-acting antivirals, different % in advanced/compensated and decompensated cirrhosis	40.9 (34.5–45.1) months	Spain	15.8 kPa	Incidence rate of events per 100 PY (95% CI) 2.35 (1.62–3.41) in advanced fibrosis 2.26 (1.73–2.94) in compensated cirrhosis 1.22 (0.55–2.72) in decompensated cirrhosis	Berenguer et al., 2024 [33]
Prospective	229	Sofosbuvir + Ribavirin (16.2%); Simeprevir/Sofosbuvir ± Ribavirin (17.5%); Sofosbuvir/Velpatasvir ± Ribavirin (11.4%); Daclatasvir/Sofosbuvir ± Ribavirin (10.0%); Elbasvir/Grazoprevir (3.5%); Ledipasvir/Sofosbuvir ± Ribavirin (20.1%); Paritaprevir/Ritonavir/Ombitasvir/Dasabuvir ± Ribavirin (20.1); Glecaprevir/Pibrentasvir (1.2%)	24 weeks 48 weeks	Italy	18.1 (±6.6) kPa	13.6 (±6.1) kPa at 24 weeks 12.5 (±6.1) kPa at 48 weeks	Nicoletti et al., 2024 [35]
Observational, cohort single-center study	57 HCV chronic kidney disease, of whom 6 in F3, 5 in F4	Ombitasvir/Parytaprevir/Rytonavir (23.7%) Ledipasvir/Sofosbuvir (52.5%) Glecaprevir/Pibrentasvir (5.1%) Elbasvir/Grazoprevir (18.6%) RBV (76.2%)	4 years after EOT	Poland	F3 (9.5–12.4 kPa) F4 (≥12.5 kPa)	Advanced fibrosis (F3–F4) patients was reduced from 19.83% (*n* = 11) to 12.3% (*n* = 7) 1 advanced fibrosis patient progressed to cirrhosis (baseline = 12.4 kPa; 4 years after EOT = 13.6 kPa)	Czarnecka et al., 2023 [36]
Prospective	Out of 43 total patients, 15 had F4, 25 had advanced fibrosis (F3–F4)	Sofosbuvir + Velpatasvir (9.3%) Sofosbuvir + Ledipasvir (37.2%) Glecaprevir + Pibrentasvir (18.6%) Paritaprevir + Ritonavir + ombitasvir + Dasabuvir (2.3%) Sofosbuvir + Velpatasvir + Voxilaprevir (4.7%) Elbasvir + Grazoprevir (27.9%)	6 to 9 months after reaching SVR	Spain	F4 (from 14 kPa and up) F3 (from 9 to 14 kPa)	Decreased fibrosis stage in 20.9% (*n* = 9) of F4 patients, in 7.0% (*n* = 3) of F3 patients	Rabell-Bernal et al., 2022 [37]
Retrospective	373 cirrhotic patients with successful HCV eradication	Sofosbuvir/Ledipasvir (+/− Ribavirin) (53.6%) Sofosbuvir (+/− Ribavirin) (13.9%) Paritaprevir/Ritonavir/Ombitasvir + Dasabuvir (+/− Ribavirin) (11.3%) Sofosbuvir + Daclatasvir (+/− Ribavirin) (7.5%) Sofosbuvir/Velpatasvir (+/− Ribavirin) (5.9%) Sofosbuvir + Simeprevir (+/− Ribavirin) (4.0%) Paritaprevir/Ritonavir/Ombitasvir (+/− Ribavirin) (2.7%) Sofosbuvir + Velpatasvir + Voxilaprevir (+/− Ribavirin) (0.5%) Elbasvir/Grazoprevir (+/− Ribavirin) (0.3%) Glecaprevir + Pibrentasvir (+/− Ribavirin) 1 (0.3%)	6 months	Italy	19.3 kPa (14.7–27)	11.6 (7.7–16.8 kPa)	Armandi et al., 2022 [38]
Longitudinal retrospective study	Patients with cirrhosis (kPa ≥ 12, *n* = 119); non-cirrhotic patients (kPa < 12, *n* = 651)	DAA treatment	Median of 11.7 months	United States	kPa ≥ 12	No significant changes in LSV over time	Mezina et al., 2022 [39]
Retrospective cohort study	172 HCV treatment responders, 102 Advanced fibrosis (F3, F4)	DAA treatment	Mean follow-up was 141 (57.9) weeks	Saudi Arabia	21.10 (11.35)	13.84 (8.47)	Alswat et al., 2022 [40]
Retrospective/prospective observational single center study	133 SVR patients, of whom 82 cirrhosis with Child–Pugh score A	DAA treatment	6, 12, 18, 24 months after EOT	Israel	15.1 kPa (range 10.5–100) using shear wave elastography	13.4 kPa (range 5.5–51) 6 months after EOT 11.4 kPa (range 6.1–35.8) 12 months after EOT 12.6 kPa (range 5.4–36) 18 months after EOT 11.5 kPa (range 5.2–16.4) 24 months after EOT	Davidov et al., 2021 [41]
Prospective	54 achieved SVR 41 in FU	Daclatasvir + Sofosbuvir ± Ribavirin (66.7%), Sofosbuvir +Ledipasvir ± Ribavirin (22.2%), Simeprevir + Sofosbuvir ± Ribavirin (9.3%), Ombitasvir/Paritaprevir/Ritonavir + Dasabuvir (1.8%)	EOT 24 weeks after EOT 1, 2 and 3 years post-treatment	Germany	32.4 (9.1–75)	21.3 (6.7–73.5) kPa at EOT 16 (4.1–75) kPa 3 years	Knop et al., 2021 [42]
Prospective	82 patients in F1/F2, 50 in F3/F4	Sofosbuvir/Velpatasvir	LSV after DAA treatment	Portugal		Out of 50 patients in F3/F4, 22.4% obtained statistically significant in t in liver fibrosis stage	Ferreira J et al. [43]
Multicenter observational study	50 HIV-infected patients with advanced HCV-related cirrhosis.	Sofosbuvir + Ledipasvir (40%), Sofosbuvir + Daclatasvir (28%), Sofosbuvir + Daclatasvir + Simeprevir (6%), Sofosbuvir + Simeprevir (20%), Ombitasvir + Paritaprevir + Ritonavir + Dasabuvir (6%)	48 weeks after DAAs treatment	Spain, United States		Significant decreases in severity scores of liver disease (LSV, HVPG, and CTP) and plasma biomarkers	Medrano et al. [44]

## Data Availability

The data that support the realization of the Figure 1 of this review are openly available in EUROSTAT. Standardised death rate due to tuberculosis, HIV and hepatitis by type of disease at https://ec.europa.eu/eurostat/databrowser/view/sdg_03_41__custom_11318007/default/bar?lang=en, (accessed on 25 April 2024).
